# *Drosophila* exoribonuclease *nibbler* is a tumor suppressor, acts within the RNA^i^ machinery and is not enriched in the nuage during early oogenesis

**DOI:** 10.1186/s41065-017-0047-z

**Published:** 2017-09-29

**Authors:** Casimiro Castillejo-López, Xiaoli Cai, Khalid Fahmy, Stefan Baumgartner

**Affiliations:** 1 Department of Experimental Medical Science, Lund University, BMC D10, 22184 Lund, Sweden; 2 Present address: Department of Molecular Epidemiology, Uppsala University, 75185 Uppsala, Sweden; 3 Present address: Department of Genetics, Ain Shams University, Cairo, Egypt

**Keywords:** *Drosophila*, Nibbler, Tumor formation, Nuage

## Abstract

**Background:**

micro RNAs (miRNAs) are important regulators of many biological pathways. A plethora of steps are required to form, from a precursor, the mature miRNA that eventually acts on its target RNA to repress its expression or to inhibit translation. Recently, *Drosophila nibbler* (*nbr*) has been shown to be an important player in the maturation process of miRNA and piRNA. Nbr is an exoribonuclease which helps to shape the 3′ end of miRNAs by trimming the 3′ overhang to a final length.

**Results:**

In contrast to previous reports on the localization of Nbr, we report that 1) Nbr is expressed only during a short time of oogenesis and appears ubiquitously localized within oocytes, and that 2) Nbr was is not enriched in the nuage where it was shown to be involved in *piwi*-mediated mechanisms. To date, there is little information available on the function of *nbr* for cellular and developmental processes. Due to the fact that *nbr* mutants are viable with minor deleterious effects, we used the *GAL4/UAS* over-expression system to define novel functions of *nbr*. We disclose hitherto unknown functions of *nbr* 1) as a tumor suppressor and 2) as a suppressor of RNA^i^. Finally, we confirm that *nbr* is a suppressor of transposon activity.

**Conclusions:**

Our data suggest that *nbr* exerts much more widespread functions than previously reported from trimming 3′ ends of miRNAs only.

## Background

In eukaryotes, three main RNA^i^ pathways have received considerable attention in the past: microRNAs (miRNAs), small interfering (siRNAs) and Piwi-interacting RNAs (piRNAs) [18]. All three pathways reveal difference in their biogenesis, type of Argonaute family proteins, mode of target regulation and substrates [17]. The RNA^i^ machinery and mechanisms associated with it are evolutionarily conserved in most eukaryotic organisms, including insects [42].

During the last decade, microRNAs (miRNAs) were found to be important regulators of development, pathology and physiology of plant, as well as humans (reviewed by [22, 48]. Despite their small size of about 22 nucleotide (nt), these RNA molecules exert complex functions by binding preferentially to the 3′ untranslated region (UTR) of target RNAs to block their function. miRNAs are initially synthesized by RNA polymerase II to yield a precursor miRNA of about 70 nt length which are 5′ capped and which are also 3′ polyadenylated which subsequently folds into a structure with a partially-paired stem, a single-stranded loop and a 2 nucleotide 3′ overhang. These 3 features are characteristic for the primary miRNA.

As a next step, these primary miRNA are exported from the nucleus to the cytoplasm. There, Dicer, a RNase III processes the pre-miRNA to a 22 nt mature miRNA/miRNA* duplex [20, 27]. Subsequently, miRNA duplexes are assembled in a complex with Argonaute (Ago) to form the precursor RNA-induced silencing complex (RISC) [11]. This complex formation appears to be uncoupled from the synthesis of the miRNA. It is also within the RISC complex where one of the strands is chosen as the active silencing complex. Finally, the active miRNA within RISC binds preferentially to the 3′ UTR of their target mRNAs which leads either to repression of the transcription or by inhibiting its translation [14].

When measuring the length of the end of the 3′ overhangs, it was noted that there was an unusually high heterogeneity within the per-miRNA molecules which was ascribed to a sloppy mode of action of RNase III. This necessitated to postulate the presence of yet another enzyme that would account for the precise outcome of the 2 nt overhang. In 2011, two groups presented Nibbler (Nbr) protein in *Drosophila* as a candidate enzyme belonging to the class of exoribonucleases which likely represented the missing link [23, 32]. in vitro assays showed that Nbr trims many miRNAs to a 22 nt product, when bound to Ago [23], exemplified on a preferred target, miR-34 [23, 32] which itself exists in several isoforms. In absence of *nbr*, all smaller isoforms of miR-34 are lost [32], indicating that Nbr has a specific function on trimming miR-34, but it would not exclude that *nbr* would have a broader set of targets. Interestingly, Nbr was predicted to contain no RNA-binding activity, therefore, it was suggested that Ago would exert this job, and only the binding of Nbr to Ago in an complex would allow to act on miRNAs. *nbr* flies were first reported to be semi-lethal and sterile [23, 32]. Later *nbr* flies were found to be viable, but showing accelerated age-related effects [15, 24, 49].

Recently, research on the Piwi protein, a protein functionally and structurally close to Ago, and the associated *piwi* pathway furthered our understanding on the mechanisms of the biogenesis of small interference RNAs [24]. The *piwi* pathway and the associated piRNAs have mainly been studied in *Drosophila*. piRNAs are 23–29-nt small RNAs expressed predominantly in the oocyte [6, 34]. Concomitantly, piRNAs were discovered as master regulators to repress transposable elements (TEs) in *Drosophila* as well as in mice, rats, nematodes, and zebra fish [4, 19, 21, 31, 37, 39, 47]. It appears that there are thousands of distinct piRNA sequences present in the genomes of *Drosophila* [4]. To date, no structural or sequence similarity between the sequences of different piRNAs was found, except for a stronger bias for uracil in the first nucleotide [4]. In *Drosophila*, piRNAs recognize their targets, which predominantly are mRNAs of TEs through perfect or nearly perfect antisense matching. Hence, interfering with the *piwi* system may change the activity of transposon which may have deleterious effects on the organisms. piRNAs undergo several steps of maturation including formation of the primary piRNAs which are loaded onto Piwi [28]. As a further maturation process, the “Ping-Pong” cycle, reported to occur in the nuage of *Drosophila* germ cells [4], amplifies secondary piRNAs and thereby silences targets [12, 43]. Due to the amplification of the piRNAs, it is thought that the process consumes transcripts of TEs, thereby leading to a silencing of TEs. Conversely, interfering with the Ping-Pong cycle has likely the opposite effect, i. e. TE transcripts are present at unusually high levels. This in turn increases the probability of TEs to insert into developmentally-important genes or tumor-suppressor genes which may have deleterious effects such as generating cancer in tissues which otherwise would not happen if the regulation of TE activity was in balance.

Given the importance of *nbr,* little information is available as to the overall function of *nbr* for development or cellular mechanisms in a broader context. We therefore thought to shed some light onto possible mechanisms. Instead of using classical mutants where information on the function is very limited, we used inducible *nbr* RNA^i^ and dominant-negative versions of Nbr, and employed the GAL4/UAS over-expression system [3]. Using these approaches, we disclose hitherto novel functions of *nbr* in (i) regulating TE activity, and in (ii) suppressing tumors. Moreover, we show that Nrb is expressed very early during oogenesis and that *nbr* is involved in regulating small interfering RNA (siRNA) activity. Taken together, our data suggests that *nbr* reveals a broader involvement in regulative cellular processes than just trimming specific miRNAs.

## Methods

### *Drosophila* stocks

All transgenic stocks were obtained using conventional transformation techniques [36] and were maintained as balanced stocks. *MS1096-GAL4*, *apterous-GAL4*, *tubulin-GAL4*, *paired-GAL4*, *en-GAL4* and *UAS-nbr* were obtained from the Bloomington stock center. *UAS-Dg* ^*i*^ flies were obtained from Martina Schneider [40].

**Fig. 1:**
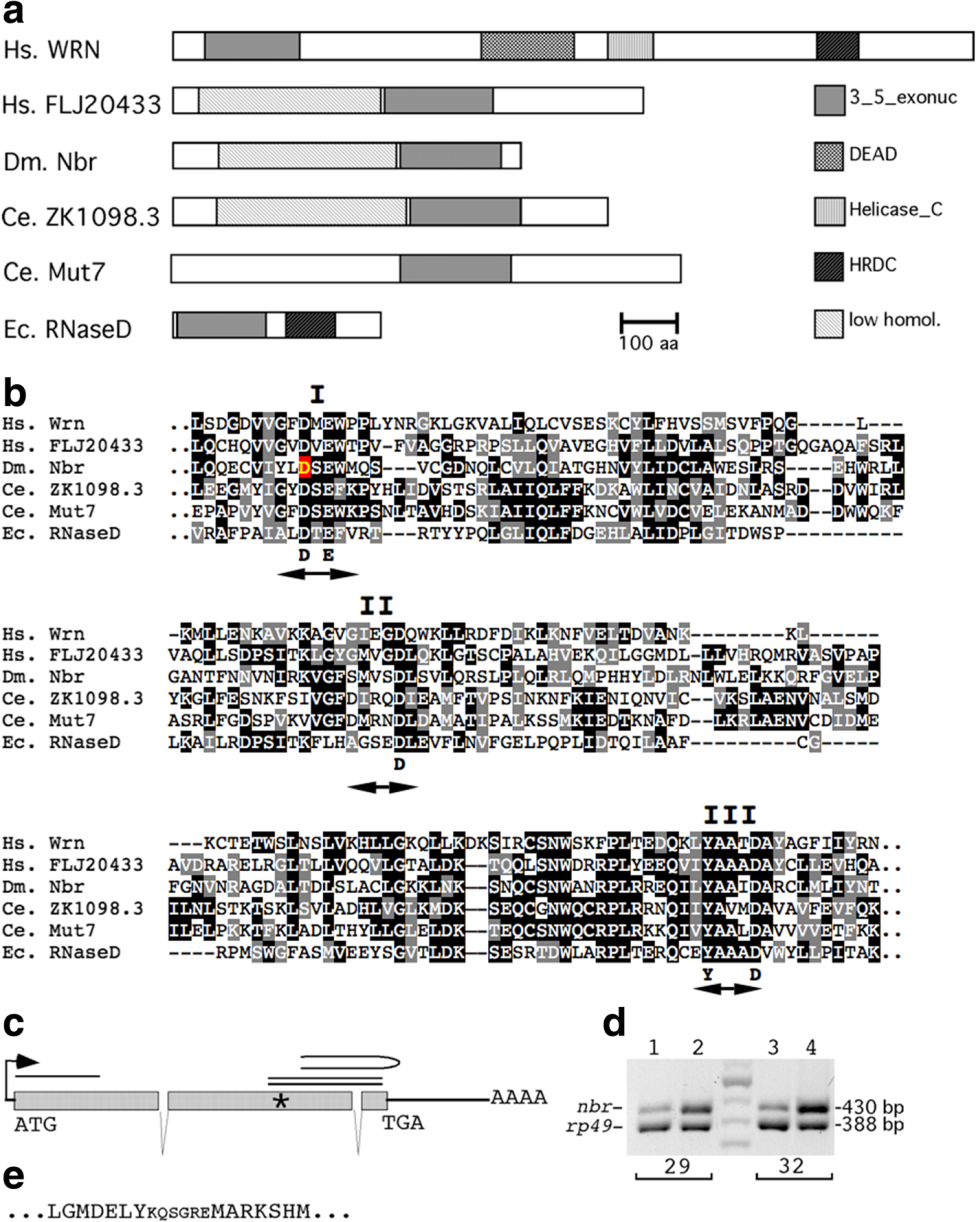
Nbr harbors a exonuclease domain and is conserved from worms to humans. **a** schematic presentation of some 3′-5′ exonuclease-containing proteins, drawn to scale. From top: human Werner syndrome protein (accession number L76937.1), human FLJ371119 mRNA (accession number AK094438.1, corrected for some sequencing errors, based on genomic DNA), *Drosophila melanogaster nibbler* (*nbr*), (CG9247, accession number NM_136250.2), *Caenorhabditis elegans* ribonuclease-like protein ZK1098.3 (accession number NM_066703.1), *Caenorhabditis elegans* Mut7 protein (accession number NM_066704.1) and *E. coli* RNase D (accession number X07055). Identified domains are indicated on the right part and comprise the 3′-5′ exonuclease domain, the DEAD box, the Helicase C domain, the HDRC domain and a low homology region common to CG9247, human FLJ20433 and *C. elegans* ZK1098.3. Note that human FLJ20443 and *Caenorhabditis elegans* ZK1098.3 are likely *nbr* orthologues as they also contain a low-homology region (shaded oblique) common to all Nbr proteins. **b** Amino acid sequence alignment of the catalytic part of the 3′-5′ exonuclease domain of the 6 proteins in Fig. 1a. Subdomain I- III nomenclature taken from [53]. Identical amino acids appear black, conservative changes appear in grey. Critically conserved amino acids appear in bold under the alignment. The critical amino acid D of subdomain I that was exchanged to N in Fig. 2 is highlighted in red/yellow. **c** Exon-intron structure of *nbr*. Gray boxes represent exons. The line above exon 1 represents the probe used for in situ hybridization, as well as for the product of the RT-PCR for silencing quantification. The double line indicates the dsRNA fragment used for silencing in S2 cells. The hairpin line above indicates the dsRNA generated in the RNA^i^ transgenic lines. The asterisk represents the position of the aspartic acid in domain I mutated in *UAS-EGFP-Nbr-N* construct. **d** Silencing effect of transgenic RNA^i^ embryos. Agarose gel electrophoresis of the RT-PCR products amplified for 29 cycles or 32 cycles. Genotypes: *UAS-nbr* ^*i*^ *37*/*UAS-nbr* ^*i*^ *37*; *tub-GAL4*/*TM3* (lanes 1 and 3); and *UAS-nbr* ^*i*^ *37*/*UAS-nbr* ^*i*^ *37; +*/*TM3* (lanes 2 and 4). Amplification of *nbr* generates a 430 bp band (see Fig. 1c), compared to the 388 bp band of the internal control from *ribosomal protein 49* (*rp49*). **e** Deduced amino acid sequence of the boundary of EGFP-Nbr used in the over-expression analysis of Fig. 2. Left side C terminus of EGFP (capital letters), middle linker region (small letters), right side N-terminus of Nbr (capital letters)

The *Drosophila* Gene Collection clone GM01690 containing the complete ORF of *nbr* was used to generate the vectors *pUAST-EGFP-nbr-D* expressing the wild-type Nbr protein fused to EGFP, and *pUAST-EGFP-nbr-N* expressing the inactive Nbr protein fused to EGFP. Mutation of the catalytic domain was done by conventional inverse PCR using primers (5′-tcatatacctgAattctgaatggat-3′) and (5′-atccattcagaatTcaggtatatga-3′) to produce the amino acid substitution D435N (colored D in Fig. 1b). Both coding sequences were amplified with primers 5′-GGGCGGCCGCGAAATGGCACGCAAG-3′ and 5′-CCTCTAGAGGCCAGTTCCTCAATC-3′. After restriction with Not I/Xba I, the amplified products were ligated in frame with EGFP, generating the vectors *pUAST-EGFP-nbr-D* and *pUAST-EGFP-nbr-N*. Sequencing of the constructs was performed before the establishment of transgenic lines.

### RT-PCR

Total RNA was isolated from ovaries or adult males and females using TRIzol (Invitrogen) and treated with DNase I (Ambion) to remove DNA. First strand cDNA was prepared using the SuperScript II reverse transcriptase kit (Invitrogen) according to the manufacturer’s instructions with 500 ng total RNA and 50 ng random hexamer primers in a 10 μl reaction.

The primers for amplification of *copia* were: (5′-ATTCAACCTACAAAAATAACG-3′) and (5′-ATTACGTTTAGCCTTGT-3′), producing a product of 438 bp. The primers for amplification of the control, the ribosomal protein-encoding gene *rp* *49* were: (5′-GACCATCCGCCCAGCATACAGGC-3′) and (5′-GAGAACGCAGGCGACCGTTGG-3′) producing a product of 388 bp. In order to compensate for the distinct abundance of transcripts, primers for *copia* were used at 200 nM and for *rp49* at 40 nM.

### In situ hybridization

Riboprobes were generated using a DIG-labeling kit (Roche). Two templates were amplified from cDNA and cloned into pBS (KS). The 5′ template including the signal peptide sequence of *nbr* was amplified with primers (5′-ATGGTACCTCGCAATGAGTGATTTAC-3′) and (5′-TATGGATCCTGCAGTTGGTTCTCTAGT-3′) generating a 467 base pair probe. The 3′ template from the cytoplasmic part of the gene was amplified with the primers, (5′-ACAAGTCGTCGTACAAGGA-3′) and (5′-GACCACCATTCTTGTTTGTAGGCA-3′) generating a 343 base pair probe. The procedure for in situ hybridization was carried out according to [46]. A sense probe was used as a negative control.

### Generation of antibodies and immunohistochemistry

A NH2-terminal peptide CNFDATLDAKAEEFFKLFREKWNM comprising aa 46-69 of Nbr was used to immunize a rabbit. Crude serum from the 2nd bleed was used in all experiments. For *Drosophila* whole-mount staining, a Nbr monoclonal antibody [24] was used at 1:100 and detected using 2nd antibodies coupled to Alexa 555. For all immunofluorescence pictures, a Zeiss LSM 710 was used. For super-resolution recording, an Airy-Scan (Zeiss™) assembly was used in combination with a 63× lens. For Western analysis, embryos from the cross of the *paired (prd)-GAL4* > *UAS-nbr* strain (Bloomington stock #16587) were used for 4-8 h extracts which, together with a 4-8 h wild-type extract of similar protein concentration were separated on a 10% PAGE and probed with the Nbr antiserum at a 1:2000 dilution.

### RNA interference in cells

S2 cells were propagated in 1× Schneider’s *Drosophila* medium (GIBCO), supplemented with 10% FBS, 50 units/ml penicillin, and 50 μg/ml streptomycin at 27 °C. dsRNAs were produced from amplified DNA templates using a MEGASCRIPT T7 transcription kit (Ambion) following the protocol of [10]. DNA templates were amplified with primers containing a 5′ T7 RNA polymerase binding site (5′-TAATACGACTCACTATAGGGAGACCAC-3) followed by sequences specific for the targeted genes. The following primers were used: *nbr* (5′- TAATACGACTCACTATAGGGAGACCACaggagtgcgtcatatacctg-3′) and (5′- TAATACGACTCACTATAGGGAGACCACgcgttcaatgagcgtgttg-3′); *GFP* (5′- TAATACGACTCACTATAGGGAgaccaccctgacctacggc-3′) and (5′- TAATACGACTCACTATAGGGagaccacgaactccagcaggacc-3′) and mock-lacZ (5′- TAATACGACTCACTATAGG-3′) and (5′- TAATACGACTCACTATAGGGAGACCAccgaactgagatacctacagc-3′), amplifying the 838 bp sequence of the vector pBluescript SK downstream of the T7 RNA polymerase binding site that includes the lacZ alpha gene. dsRNA products were DNase- treated, ethanol-precipitated and resuspended in DEPC water. The dsRNAs were analyzed by agarose gel electrophoresis to ensure that single bands of the expected size were present. S2 cells were transfected using FuGENE 6 (Roche) in 3 cm dishes at 50%–70% confluence, following the manufacturer’s recommendation. The standard transfection reaction contained 2 μg of plasmid expressing GFP (pAC-EGFP), 2 μg of dsRNA targeting GFP and 2 μg of either *nbr* dsRNA or *mock* dsRNA.

### Transgenic RNA interference construct

A 408 bp fragment at the 5′ of the *nbr* gene (Fig. 1c) was amplified from cDNA with primers 5′-TGGTACCAGTGATCTCAGTGTATTGCAG-3′ and 5′-CGGATCCTCAATCACTTAACATGGGCA -3′. After digestion with Kpn I/Bam HI, the fragment was subcloned both into pBluescript II (Stratagene) forming pKS-nbr and into pEGFP-N1 (Clontech), respectively. Inversion of the sequence was achieved by excision of the Nhe I/Bam HI fragment of the pEGFP-N1 construct and subsequent ligation with a 148 bp Sau3A linker, derived from digestion of pEGFP-N1 with Sau3A, into pKS-nbr, cut with Spe I/Bam HI*.* The 964 bp Kpn I fragment containing the inverse sequence separated by the linker was inserted into pUAST. Prior to transformation, the construct was verified by restriction analysis and sequencing.

## Results

### *Drosophila* Nibbler belongs to the exonuclease D family

In search for 3′-5′ exonuclease domain-containing proteins within the *Drosophila* genome, we came across a transcript, originally termed CG9247 by the *Drosophila* sequencing consortium (BDGP; [9], which contained a typical 3′-5′ exonuclease domain found in many proteins from worms to humans (Fig. 1a), subsequently termed *nibbler* (*nbr*) [23, 32]. Analysis of the *nbr* open reading frame (ORF) revealed a protein of 625 amino acids with two domains shared by other proteins in the animal kingdom. At the amino terminus, there is no indication of a signal peptide indicative of a secreted or a transmembrane protein, suggesting that it is an intracellular protein. The first third of the protein (amino acids 1-181) does not contain any homology to any known protein, while the second third (amino acids 182-409) contains low homology to human protein FLJ20433 and *C. elegans* nuclease ZK1098.3. The last third of the protein contains the 3′-5′ exonuclease domain which was the initial searching bait. This domain is a widespread domain found in diverse proteins such as human Werner syndrome protein [51] or *E. coli* RNaseD [52]. Due to the fact that human FLJ20433 and *C. elegans* ZK1098.3 contain a similar domain arrangement as CG9247 and because lengths of all three proteins are similar, we presume that these three proteins represent the respective *nbr* orthologues.

Analysis of the *nbr* 3′-5′ exonuclease domain revealed that the sequence homology is fairly good (Fig. 1b), in particular the absolutely preserved amino acids that are the typical characteristics of an exonuclease domain. This domain is part of a large DEDD subfamily of exoribonucleases [53], owing to the fact that they contain invariant acidic amino acids at certain position such as the aspartic acid D and the glutamic acid E within domain I (Fig. 1b) and two aspartic acids D in domain II and III, respectively (Fig. 1b). This DEDD subfamily includes the proof-reading domains of many DNA polymerases as well as other DNA exonucleases and shares a common catalytic mechanism characterized by the involvement of two metal ions [44]. Of the different members of the DEDD subfamily, the Nbr protein resembles most the RNaseD proteins which further subdivide the DEDD subfamily into the DEDDy sub-subfamily [53], due to the presence of an invariant tyrosine Y within the catalytic domain III (Fig. 1b).

### *nbr* exhibits nuclease activity

**Fig. 2:**
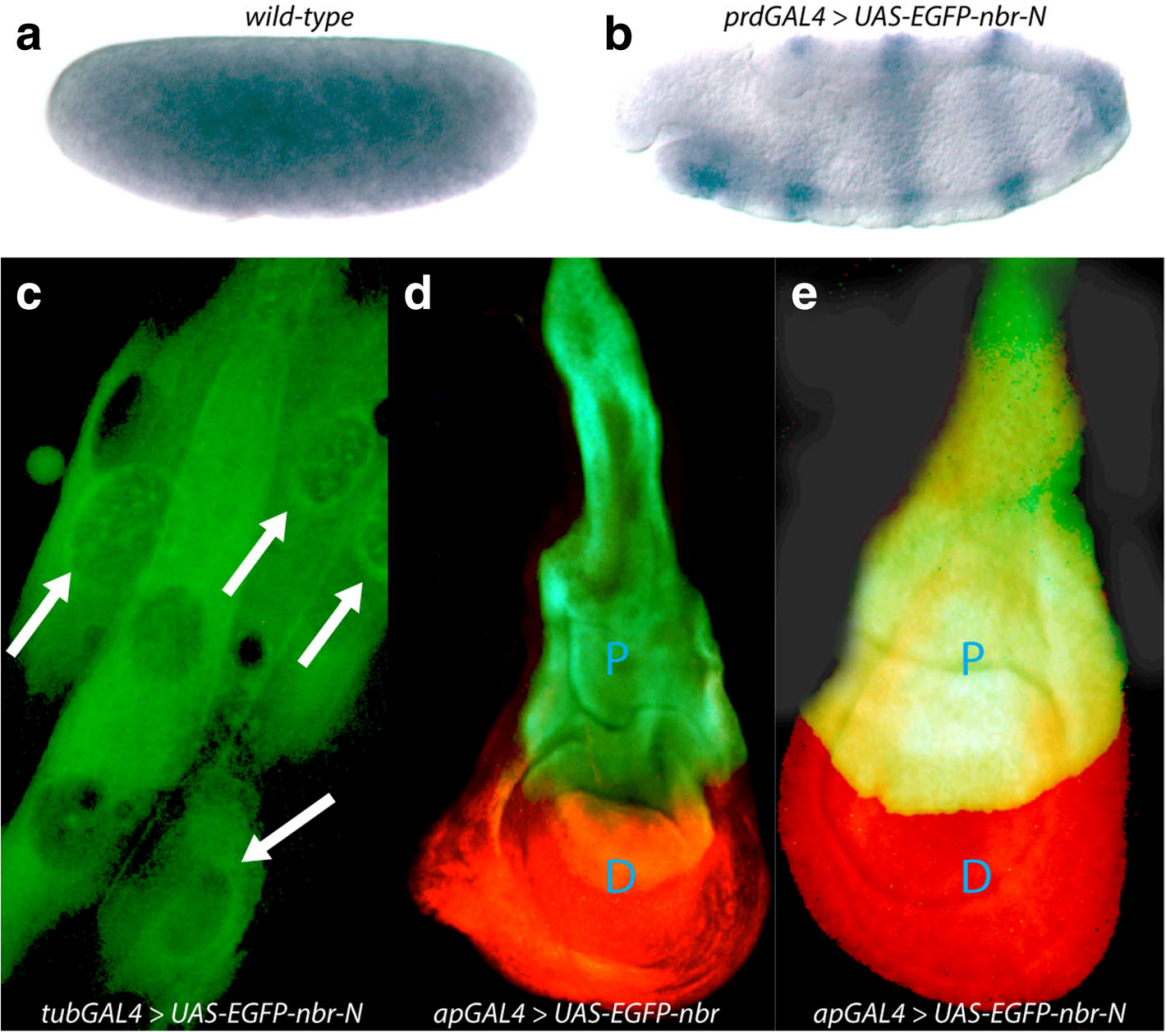
Embryonic expression, cellular location and nuclease activity of Nbr. **a** in situ hybridization of a wild type stage 2 embryo (stages are according to [7]. The transcript is maternally expressed and appears ubiquitously. **b** in situ hybridization, using the same probe as above, of a stage 11 embryo expressing the transgene *UAS-EGFP-nbr-N*, driven by *prd-GAL4*. As expected, the expression of the transgene shows a pattern in 7 stripes. Endogenous *nbr* transcripts are at low levels at this stage. **c** salivary glands cells of 3rd instar larvae expressing the EGFP-Nbr-N fusion protein from the cross *UAS-EGFP-nbr-N*, driven by *tub-GAL4*. The fusion protein is enriched at the nuage surrounding the nuclei and at lower levels also located in the cytoplasm of the cells. **d** and **e** nuclease activity of wild-type and catalytic site-defective recombinant Nbr proteins. Wing discs expressing wild-type EGFP-Nbr (**d**) and EGFP-Nbr-N (**e**) under the control of *apterous (ap)-GAL4*. Nucleic acids are stained with propidium iodine (PI, red). **d** EGFP-Nbr protein expressed in the proximal compartment (P) is an active nuclease and degrades nuclei, hence PI disappears and only EGFP remains. Note also the extensive shrinking of the P part of the wing upon nuclease activity. The distal compartment (D) is not affected, hence PI is visible. (E) Fusion protein with the disrupted catalytic domain (EGFP-Nbr-N) does not reveal any nuclease activity. Hence, PI remains and merges with EGFP to yellow color. Discs in (**d**) and (**e**) are oriented with P to the top and D to the bottom

To explore the function and activity of the exonuclease domain, we inactivated the domain through a change of the invariant amino acid D to N within the catalytic domain I (highlighted in Fig. 1b, c), and by fusing the enhanced green-fluorescent protein (EGFP) to this altered Nbr protein as an EGFP-Nbr-N fusion protein (Fig. 1e). We then used the *Drosophila* wing imaginal disc as a model tissue to test the nuclease activity of Nbr and its catalytic-dead variant, Nbr-N, by using the GAL4/UAS over-expression system [3]. We over-expressed Nbr-EGFP in the proximal part of the wing disc using the *apterous-(ap) GAL4* line which drives transgenes in the proximal compartment (P) of the wing. In *ap-GAL4 > UAS-Nbr-EGFP* wing discs, we detected strong nuclease activity in the proximal compartment of the wing disc (Fig. 2d). Upon propidium-iodine (PI) staining, we observed substantial nucleic acid (NA) degradation in the proximal compartment such that only the green color from the EGFP–Nbr fusion protein remained visible. Moreover, this NA degradation led to extensive apoptosis, demonstrated by a collapsed proximal part of the wing disc. Conversely, the distal part was not affected by the ectopic expression of Nbr and thus appeared normal. These data suggest that Nbr is an potent nuclease. Conversely, a mutant form of Nbr, Nbr-N, is unable to exert any nuclease activity upon over-expression with *ap-GAL4* (Fig. 2e). Hence, even though Nbr is overexpressed in the proximal compartment, it shows intact cells, evidenced by positive staining of PI, intact NA and the change of the merged color of PI and EGFP into yellow. Moreover, this defective nuclease activity does not compromise cell survival, therefore the shape of the proximal part of the wing disc appears normal.

### Localization of the *nbr* mRNA and protein

We employed in situ hybridization to detect the spatial transcript pattern of *nbr* in *Drosophila* whole mount embryos. *nbr* is expressed ubiquitously in the early developing embryo (Fig. 2a), suggesting a maternal deposition, also confirmed by FlyBase [1]. At stage 5, i. e. at cellular blastoderm stage, *nbr* expression drops considerably, and transcripts are only detected ubiquitously at low level during the remaining embryonic stages (FlyBase, [1]; data not shown). To ease analysis of the localization and function of *nbr*, we constructed *EGFP-Nbr* flies under control of the GAL/UAS system that allows ectopic expression of any protein [3]. To test the functionality of the *EGFP-nbr-N* transgene, we monitored the expression of the ectopic transcript driven by the *paired (prd)-GAL4* driver using in situ hybridization. As is evident from Fig. 2b, the *EGFP-nbr-N* transgene is faithfully expressed in 7 stripes, compared to the low-level ubiquitous expression of the endogenous *nbr* transcripts.

To investigate the subcellular localization of the Nbr protein, we first analyzed the EGFP-Nbr wild-type fusion protein in salivary gland cells from third instar larvae using a *tubulin (tub)-GAL4* driver line. As evident in Fig. 2c, fluorescence was detected at low levels in the cytoplasm and at a perinuclear localization, similar to the localization of Nbr in the nuage, as described by [24].

**Fig. 3:**
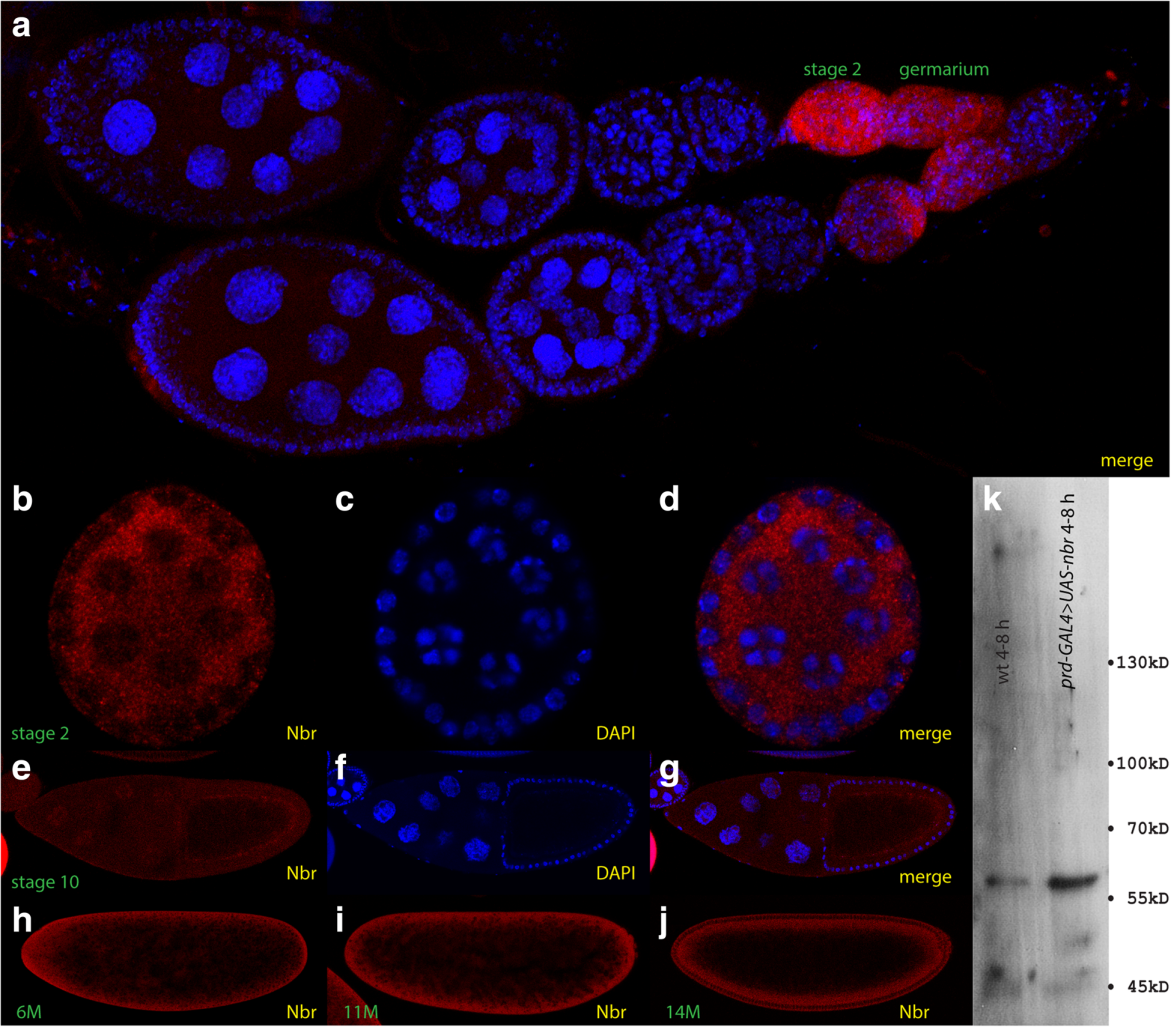
Cellular localization of Nbr and Western analysis. **a** a string of ovaries of different stages stained for Nbr (red) and the nuclear stain DAPI (blue). Nbr is expressed only in all 3 stages of the germarium and stage 2 oocytes. **b**-**d** super resolution picture of a stage 2 oocyte stained for Nbr (**b**), DAPI (**c**) and merge (**d**). **e**-**g** stage 10 oocyte stained for Nbr (**e**), DAPI (**f**) and merge (**g**). **h**-**j** staining of Nbr in embryos, nuclear cycle (nc) 6 embryo (**h**), nc 11 embryo (**i**) and nc 14 embryo (**j**). **k** Western analysis of wild-type 4-8 h extracts (left lane) and *prd-GAL4* > *UAS-nbr* 4-8 h extracts (right lane) probed with the *nbr* antiserum. A single band of ~60 kD is observed which is upregulated upon over-expression using the *prd-GAL4* driver (right lane)

To evaluate the nature and appearance of the protein, we used an antiserum which was directed against a peptide residing at the NH2-terminal part of the Nbr protein. On Western analysis, a 60 kD band was readily detected in wild-type 4-8 h extracts (Fig. 3k) in agreement with other reports [15]. The intensity of the 60 kD band was substantially increased when Nbr was over-expressed using *prd-GAL4* driving an *UAS-nbr* construct during an identical time interval and upon equal protein amount loaded (Fig. 3k).

To monitor the subcellular expression, we stained *Drosophila* tissues using a monoclonal antibody against Nbr [24] along with a nuclear stain, DAPI. We first focused on oogenesis, since *nbr* was reported to play a critical role during this stage [15, 24]. We detected the protein during the earliest stages in the germarium and in stage 2 oocytes only (Fig. 3a), while all subsequent stages were devoid of Nbr. Using super-resolution microscopy aimed to detect Nbr localization subcellularly and in the nuage [24], the Nbr protein was detected uniformly in the cytoplasm of stage 2 oocytes (Fig. 3b). This localization data are inconsistent with the data of [24]) who reported staining in the nuage surrounding the early nurse cells, but otherwise devoid of any other cellular localization. Moreover, our subcellular Nbr localization data do not entirely fit the localization pattern of EGFP-Nrb (Fig. 2c), however, the data was recorded in two different tissues, the salivary gland vs. the oocyte. Moreover, both the data by [24] and the data of Fig. 2c were based on the use of GFP-Nbr constructs rather than true antibody detection. Hence, we propose that the Nbr-EGFP fusion proteins tend to accumulate in the nuage and thus lead to a misinterpretation of the location where Nbr acts. On the other hand, our antibody data fit another report that revealed Nbr ubiquitous expression in the cytoplasm of oocytes [15] using a Nbr-HA fusion protein and anti HA staining, hence the localization does not include an EGFP-tag, but rather a small and binding-neutral HA-tag. In summary, there are marked differences between the EGFP-Nbr localization and the true antibody staining which question the nuage staining by [24].

Later during oogenesis, i. e. during stage, Nrb was detected at low levels at the cortex of the oocyte (Fig. 3e) which persisted during later stages of oogenesis (data not shown). In freshly-laid embryos, the cortical pattern was particularly pronounced (Fig. 3h), due to the strong maternal loading of *nbr* mRNA (Flybase; [1]. The cortical pattern persisted during early nuclear stages, including nc 11 embryos (Fig. 3i) when the majority of the nuclei have reached the periphery of the blastoderm. At cellular blastoderm, this cortical staining persisted (Fig. 3j), and Nrb was detected on the basal as well as on the apical side of the nuclei, however, at low levels, leaving the nuclei free of Nbr staining.

### Down-regulation of the *nbr* nuclease activity can lead to tumor formation.

**Fig. 4:**
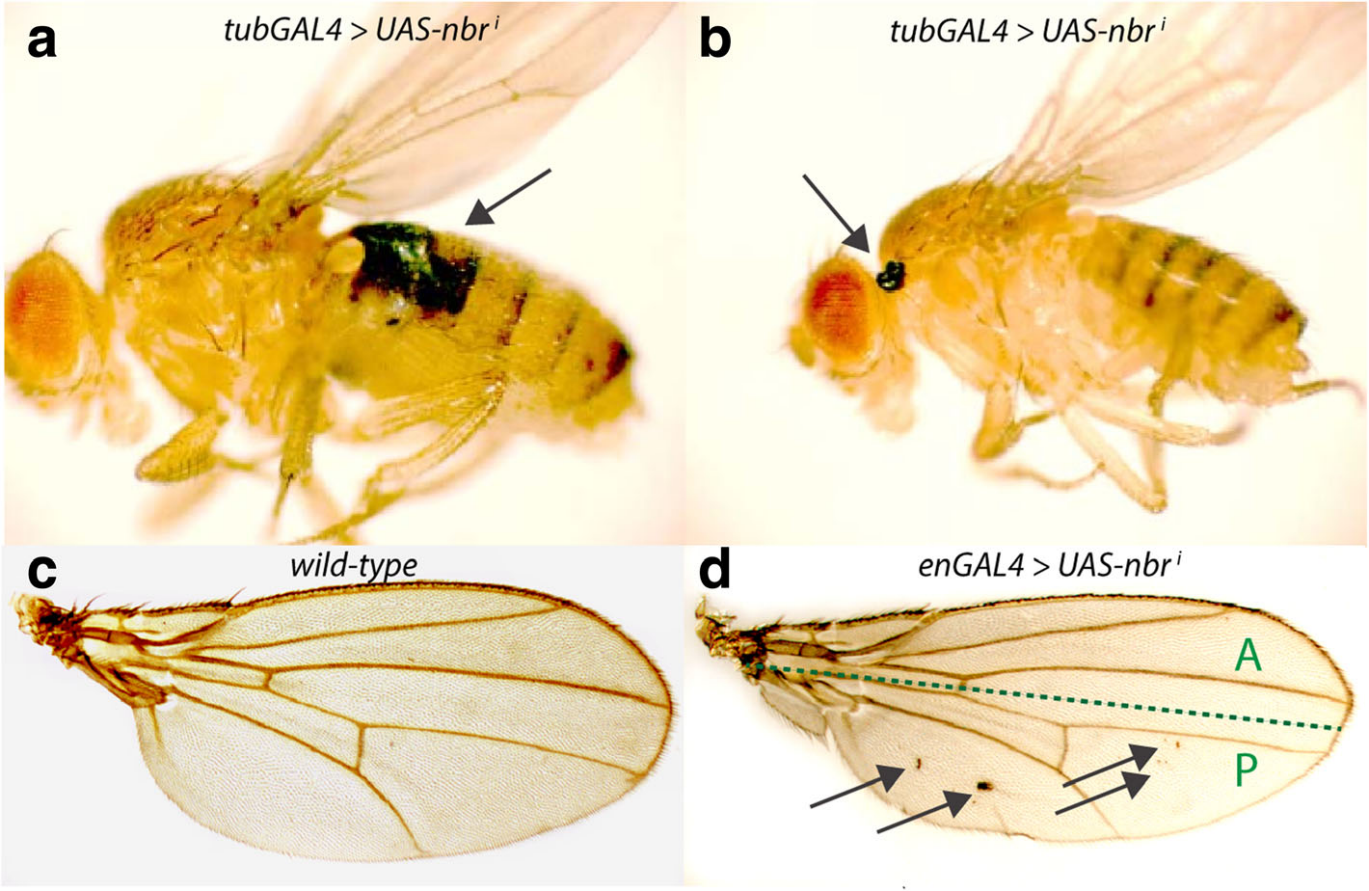
Increased tumor formation in *nbr* knock-downs. Melanotic (**a**) and epithelial (**b**) tumors (arrows) in RNAi transgenic adult females upon silencing of *nbr*, using *UAS-nbr* ^*i*^ *37*/*UAS-nbr* ^*i*^ *37*; *tub-GAL4*/*TM3*. **c** Wild type and (**d**) *nbr*- silenced wing the posterior compartment, using *UAS-nbr* ^*i*^ *19*/*UAS-nbr* ^*i*^ *19*; *en-GAL4*/*en-GAL4*. Tumors are found primarily in the posterior compartment. The green dashed line represents the anterior/posterior (A/P) compartment boundary

To determine the function of *nbr* for development, we used a GAL4/UAS-inducible knock-down strategy [29]. To this end, a hairpin cDNA construct (Fig. 1c) was cloned into the pUAST vector [3] and transgenic lines were established. The knockdown activity of this construct was measured in a reverse transcriptase-PCR approach and was found to lead to a 60% reduction of *nbr* mRNA (Fig. 1d). Interestingly, one of these lines, *UAS-nbr* ^*i*^ *37* showed incidences of melanotic tumors (Fig. 4a) or solid tumors (Fig. 4b) (arrows), when crossed to an ubiquitously-expressed *tub-GAL4* driver line. Due to the widespread occurrence, we then sought to limit the occurrence of cancer to well-defined areas. To this end, an *engrailed (en)-GAL4* driver line which is active in posterior wing compartments (P, Fig. 4d) was crossed to *UAS-nbr* ^*i*^ *19*, and the wings of the progeny were examined. We found small tumors exclusively in the posterior compartments of the wings (Fig. 4d, arrows), consistent with *en* expression in the posterior compartment of the wing. Thus, it appeared that down-regulation of *nbr* increased the incidence of cancer. We reasoned that the activity of Nbr might be needed for control of cell divisions or for control of activity of transposons which randomly affect the activity of cell cycle regulators. Alternatively, the increase of incidences of melanotic tumors in *nbr*-depleted tissues could be a consequence of the impairment of normal aging processes as previously reported by [15].

### Knockdown of *nbr* reduces the effect of RNA^i^

Next, we pondered if *nbr* might be involved in mechanisms of the RNA^i^ machinery. For this reason, we set up two parallel assay systems, a cell-culture based system and a transgenic fly approach to test if *nbr* is involved in RNA^i^. *Drosophila* Schneider cells S2 were transfected with a reporter plasmid driving EGFP by an actin promoter. In parallel, two *RNA* ^*i*^, one against the RNA of *EGFP* and another one against the RNA of a *mock* gene, *LacZ*, were applied simultaneously.

**Fig. 5:**
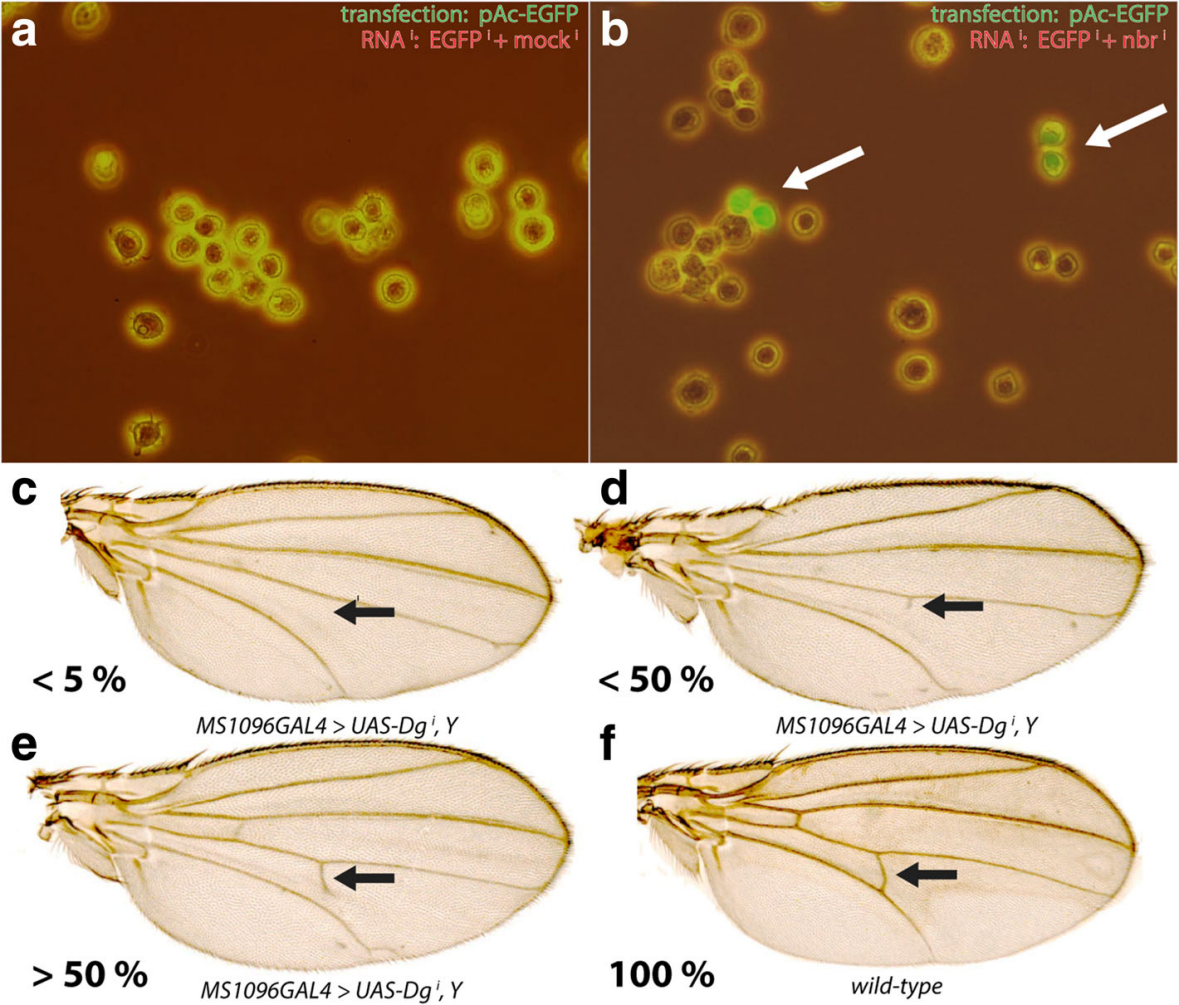
*nbr* knockdown reduces the silencing effect of RNA interference. **a** and **b** Reduction of the silencing effect of *nbr* in S2 cells. **a** cells transfected with the reporter plasmid pAc-EGFP and RNA^i^ against EGFP and mock RNA^i^. Expression of EGFP is completely abolished. **b** When *mock RNA* ^*i*^ is replaced with *nbrRNA* ^*i*^, expression of the EGFP reporter gene is increased in a number of cells. **c**-**f** phenotypic classes of males flies expressing dsRNA against *Drosophila dystroglycan* (*Dg,* CG18250) related to the occurrence of the posterior cross vein (PVC) in wings, divided into 4 classes of decreasing severity: (**c**) <5%, (**d**) 5-50%, (**e**) 50-100% and (**f**) 100% = wild-type. In wings of *Dg*-knockdown flies, PVCs extending more than 50% are observed in ~53% of the wings (see Table 1). In wings of doubly *Dg, nbr* knockdown flies, this number is increased to ~73%, demonstrating that the RNA^i^ activity of *Dg* is suppressed by lowered activity of *nbr*

The expression of the GFP transgene is complete suppressed when the cells are co-transfected with *RNA* ^*i*^ against *EGFP,* or *RNA* ^*i*^ against *EGFP* and *RNA* ^*i*^ against the *mock* gene *lacZ* (Fig. 5a). The efficiency of RNAi is reduced when the *mock RNA* ^*i*^ is substituted with the *RNA* ^*i*^ against *nbr* (Fig. 5b), demonstrating that suppression of *nbr* suppresses the RNA^i^ machinery.

When then assayed *nbr* function using an in vivo system and took advantage of the fact that knock-down of the *Drosophila dystroglycan* (*Dg*) gene affects the formation of the posterior cross vein (PCV) in wings of the progeny of *MS1096-GAL4* > *UAS-Dg* ^*i*^ flies (Fig. 5c, arrows) [13]. In this genetic combination, the variability of loss of PCV tissue ranged from total absence to slight reduction, as indicated in Table 1. In ~30% of the flies, a complete reduction of the PCV was observed (Fig. 5c), while in ~16% of the cases, the PCV was present half-way (Fig. 5d). In about 53% of the cases, more than half of the PCV was present (Fig. 5e). However, when *nbr* was knocked-down using *UAS-nbr* ^*i*^ *37* flies, the loss of PCV was greatly ameliorated and only ~15% showed complete PCV loss (Table 1). More than 72% of the flies showed more than half of the PCV present, compared to 53% in *UAS-Dg* ^*i*^ (Table 1). An unrelated gene, GC3505, a serine protease not involved in wing development (C. Castillejo-Lopez, unpublished), did not significantly alter the loss of PCV upon knock-down in the same genetic background, as compared to the *UAS-Dg* ^*i*^ reference flies (Table 1). This data demonstrated that lowering *nbr* activity caused a reduction of the effect of RNA^i^. It also suggested that the exonuclease activity of *nbr* was tightly linked to the process of RNA^i^.

**Table 1:** Occurrence of posterior cross vein (PCV) in *Dg* knockdown flies, assayed together with 3 different genetic backgrounds

Genotype	PCV < 5%	PCV < 50%	PCV > 50%	Total wings
*MS1096*-*GAL4* > *Dg* ^*i*^/*Y*; *ftl* ^*i*^/+	30.1%	16.5%	53.4%	402
*MS1096*-*GAL4* > *Dg* ^*i*^/*Y*; *3505*-*2a* ^*i*^/+	37.5%	7.6%	54.9%	662
*MS1096*-*GAL4* > *Dg* ^*i*^/*Y*; *nbr* ^*i*^ *37*/+	14.9%	12.4%	72.7%	442

Allelic classes are subdivided into 3 classes: those flies that show <5% PCV occurrence, those <50% PCV occurrence and those with >50% occurrence. As read out system, a *MS1096-GAL4*-driven *Dg* knockdown construct [13] was used. This construct creates loss of PCV to various extent (Fig. 4c). Lane 1, an unrelated *ftl* gene [8]. Lane 2, *CG3505*-*2a* ^*i*^ (unpublished data). Both unrelated genetic backgrounds were used as a negative controls. Lane 1 and 2 show that 54% of all flies have 50% or more PCV tissue present. Lane 3, in doubly *ds-nbr*; *ds-Dg* flies, this value increased to 73%, indicating that lowering *nbr* activity suppresses the knock-down activity on *Dg*, suggesting that *nbr* interferes with the RNA^i^ machinery

### *nbr* affects the levels of transposon RNA intermediates

**Fig. 6:**
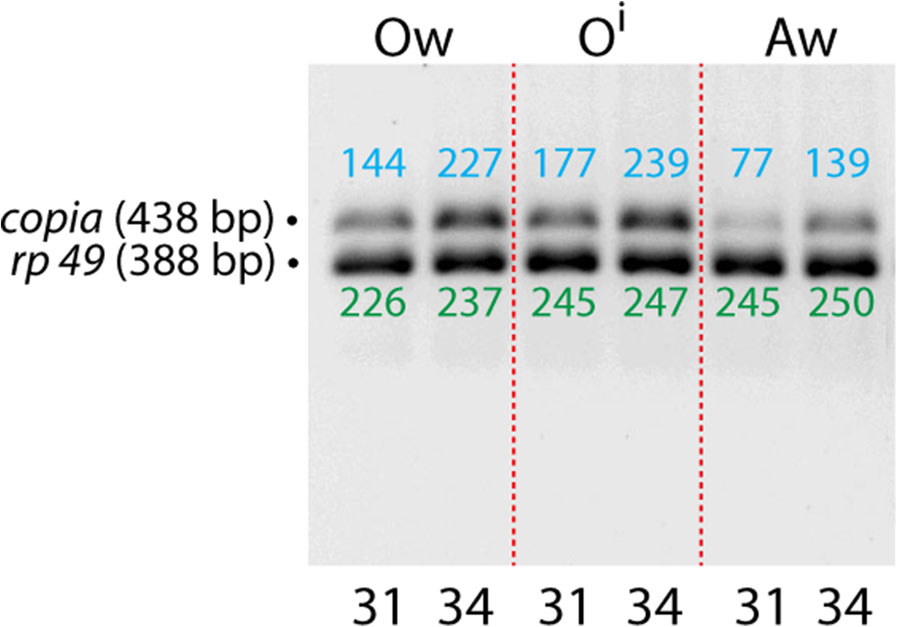
*nbr* ablation increases retro-element RNA intermediates. Semi quantitative RT-PCR of total RNA from ovaries of wild type females (Ow), transgenic RNA^i^ *UAS-nbr* ^*i*^ *37*/*UAS-nbr* ^*i*^ *37*; *tub-GAL4*/*TM3 Sb* ovaries (O^i^) and as a further control of total RNA of whole wild type adult females (Aw), assayed for the *copia* TE showing up as 438 bp fragment. PCR reactions were 31 and 34 cycles, as indicated at the bottom. Blue numbers above the bands indicate values of the optical intensity of the bands upon optical measurement, shown in arbitrary units. The RNA levels of the *ribosomal protein 49* (*rp49*) gene were taken as internal control showing up as a 388 bp band, whose optical intensities are indicated as green numbers below the bands. The length of the target genes is indicated to the left

The close similarity of Nbr to *C. elegans* Mut-7 (Fig. 1), a gene involved in TE silencing, and the elevated levels in occurrence of tumors in Fig. 4 prompted us to investigate if theses tumors were caused by elevated levels of transposon activity. We reasoned that the increased TE hopping frequency into genes regulating cell cycles would ultimately lead to an increase of incidences of tumors. It is well known that the *Drosophila* germ line is constantly exposed to high activity of transposons [33, 38, 50], hence, mechanisms must exist that limit the hopping of transposons. We therefore investigated the activity of TEs within the female germ line and compared the intermediate RNA level of the *copia* transposons in wild-type and *nbr* knock-down ovaries. As is evident in Fig. 6, the level of *copia* RNA intermediates is 33% higher in *nbr* ^i^ ovaries compared to wild-type ovaries after 31 PCR cycles, after calibration with a reference gene, *rp 49*. However, after 34 nuclear cycles, the PCR reaction is close to saturation and only a 1% increase was observed, after calibration with *rp 49*. These semi-quantitate data suggests that *nbr* serves as a suppressor of *copia*. Moreover, a comparison between tissues revealed that the relative rate of transposition is considerably higher in ovaries, compared to that of whole adult females (Fig. 6, Aw).

## Discussion

Using in vivo studies and by exploiting the GAL4/UAS system in *Drosophila,* we have analyzed the function of the Nibbler protein for development and for cellular mechanisms. We have undisclosed novel functions of this protein which suggest more wide-spread functions than hitherto anticipated.

Data from Fig. 2 indicate that Nibbler possesses a general nuclease activity and is probably more widely involved in cellular activities than only involved in trimming small RNA ends [23, 32]. This result is not surprising, as the protein possesses an exonuclease domain, however, this report shows for the first time that Nbr shows a broader involvement in trimming mRNAs. Discussed as a possibility by [15] that Nbr affects the length of not only miRNAs, but also that of *piRNAs*, it was speculated that Nbr could potentially trim the 3′ ends of a much broader species of RNA substrates, including other long and short noncoding RNAs and mRNAs. However, presumed to be instructive for the *piRNA* pathway, *nbr* has received little attention in the context of general function of RNA trimming.

Nbr appears to control the expression levels of TEs, as exemplified by *copia* in Fig. 6. As noted by [49], *copia* expression is also increased in *nbr* mutants which is in line with our observation (Fig. 6) that *nbr* controls the activity of TEs. Whether the regulation is direct or indirect via the *piwi* pathway which is involved in regulating the levels of TEs in germ cells, in currently unknown, but we favor an involvement of the *piwi* pathway.

Our data on Fig. 4 indicated that knock-down of *nbr* provokes the formation of tumors. Our current hypothesis is that ablation of *nbr* increases the rate of transposition. In these cases, the *piRNA* pathway is probably not involved, as the pathway is restricted to germ cells and extremely little *piwi* expression was observed during larval stages (Flybase; [1]. Instead, we envision that *nbr* is involved in the *miRNA* pathway, by controlling any of the multi-isoform miRNAs that are expressed during larval and pupal stage [23, 32]. These are then thought to control genes regulating cell-cycles or cell-cycle check points.

Since a while, it is known that miRNAs are involved in tumorigenesis, where the focus is mainly in humans [25, 30, 45]. To date, in *Drosophila*, only a handful of miRNA genes are known to be involved in the formation of cancer. One of them is the *bantam* gene, identified by a conventional gain of function screen which constitutes a miRNA gene that positively regulates cell proliferation and suppresses apoptosis – two features typical of oncogenes [5, 26]. However, with the advent of the availability of systematic studies by applying inducible *Drosophila* miRNA transgenes, scores of uncovered of surprisingly specific, dominant phenotypes were discovered [2, 16]. These surveys suggest that miRNA gain of function may generate diseases much more frequently than miRNA loss of function.

Our sensitive assay on *Dg*-RNA^i^-mediated depletion of the posterior cross vein (PCV) of Fig. 5c-e and Table 1 confirms a direct involvement of *nbr* in RNA^i^-mediated gene silencing. If *nbr* is reduced, the activity of the RNA^i^ machinery is weakened and depletion of PCV structures is reduced substantially. Likewise, we could confirm the mechanistic action of *nbr* in cell culture assays which revealed that effect of RNA^i^ was weakened when *nbr* activity was compromised (Fig. 5b). Hence, for the first time, we can demonstrate that *nbr* is involved in patterning processes involving whole tissues. Moreover, our data demonstrate that *nbr* is part of a general RNA^i^ machinery and not just involved in trimming selected miRNAs [23, 32].

In the past, there has been considerable disagreement as to the localization of Nbr [15, 24]. The nuage-based localization of Nbr [24], based on its involvement in the *piwi*-pathway was born by the necessity to reveal colocalization of Nbr with Aub/Ago3 in the nuage, and to adapt its localization to fit the model. Arguably, for localization studies, it is not recommended to use a fusion protein involving EGFP as in [24], as it can lead to substantial localization artefacts due to oligomerization [35, 41]. Consistent with this observation was the fact that our EGFP-Nbr fusion protein, apart from general cytoplasmic localization, also showed perinuclear localization in 3rd instar salivary glands (Fig. 2c), similar to the EGFP-Nbr localization reported in the nuage [24]. In fact, there is not an immediate necessity to describe Nbr enrichment in the nuage as claimed by [24]. Instead, it would have sufficed to imply ubiquitous Nbr localization which also includes localization in the nuage, in order to fulfill the model. This argument was put forward by [15] who observed ubiquitous Nbr localization within oocytes as well, however, their expression profile differed slightly from ours and Nbr was reported to be ubiquitously expressed beyond oocyte stage 2. Ubiquitous Nbr expression rather than accumulation in the nuage also makes sense from another perspective: Given the wide-spread involvement [15, 49] (this report), the function of Nbr is needed in the whole cytoplasm and not just in the nuage.

## Conclusions

We have shown that *nbr* is a tumor suppressor gene, and that the protein is involved in the RNAi machinery and controls the levels of transposons. Nbr is expressed only during a short time window during oogenesis and is not enriched in the nuage. Hence, we have described novel functions of *nbr* that go beyond from what was expected from previous knowledge on the mode of action of *nbr*. The ubiquitous localization of Nrb in oocytes necessitates a further careful analysis as to the mode of action of this protein. While it is not excluded that indeed it localizes to the nuage, it is not the sole subcellular location where Nbr resides which asks for further functions of this protein in other areas of the cytoplasm. Moreover, our data will encourage studies to show that Nbr is involved in many cellular processes.
